# Wound Image Quality From a Mobile Health Tool for Home-Based Chronic Wound Management With Real-Time Quality Feedback: Randomized Feasibility Study

**DOI:** 10.2196/26149

**Published:** 2021-07-30

**Authors:** Jia Zhang, Carina Mihai, Laura Tüshaus, Gaetano Scebba, Oliver Distler, Walter Karlen

**Affiliations:** 1 Mobile Health Systems Lab, Institute of Robotics and Intelligent Systems Department of Health Sciences and Technology ETH Zurich Zurich Switzerland; 2 Department of Rheumatology University Hospital Zurich University of Zurich Zurich Switzerland

**Keywords:** data quality, remote assessment, digital ulcers, scleroderma, mobile app, digital health, ehealth, mhealth, telemedicine, teledermatology

## Abstract

**Background:**

Travel to clinics for chronic wound management is burdensome to patients. Remote assessment and management of wounds using mobile and telehealth approaches can reduce this burden and improve patient outcomes. An essential step in wound documentation is the capture of wound images, but poor image quality can have a negative influence on the reliability of the assessment. To date, no study has investigated the quality of remotely acquired wound images and whether these are suitable for wound self-management and telemedical interpretation of wound status.

**Objective:**

Our goal was to develop a mobile health (mHealth) tool for the remote self-assessment of digital ulcers (DUs) in patients with systemic sclerosis (SSc). We aimed to define and validate objective measures for assessing the image quality, evaluate whether an automated feedback feature based on real-time assessment of image quality improves the overall quality of acquired wound images, and evaluate the feasibility of deploying the mHealth tool for home-based chronic wound self-monitoring by patients with SSc.

**Methods:**

We developed an mHealth tool composed of a wound imaging and management app, a custom color reference sticker, and a smartphone holder. We introduced 2 objective image quality parameters based on the sharpness and presence of the color checker to assess the quality of the image during acquisition and enable a quality feedback mechanism in an advanced version of the app. We randomly assigned patients with SSc and DU to the 2 device groups (basic and feedback) to self-document their DU at home over 8 weeks. The color checker detection ratio (CCDR) and color checker sharpness (CCS) were compared between the 2 groups. We evaluated the feasibility of the mHealth tool by analyzing the usability feedback from questionnaires, user behavior and timings, and the overall quality of the wound images.

**Results:**

A total of 21 patients were enrolled, of which 15 patients were included in the image quality analysis. The average CCDR was 0.96 (191/199) in the feedback group and 0.86 (158/183) in the basic group. The feedback group showed significantly higher (*P*<.001) CCS compared to the basic group. The usability questionnaire results showed that the majority of patients were satisfied with the tool, but could benefit from disease-specific adaptations. The median assessment duration was <50 seconds in all patients, indicating the mHealth tool was efficient to use and could be integrated into the daily routine of patients.

**Conclusions:**

We developed an mHealth tool that enables patients with SSc to acquire good-quality DU images and demonstrated that it is feasible to deploy such an app in this patient group. The feedback mechanism improved the overall image quality. The introduced technical solutions consist of a further step towards reliable and trustworthy digital health for home-based self-management of wounds.

## Introduction

### Background

Chronic wounds do not heal within the expected time and can lead to severe complications if not treated appropriately. Therefore, chronic wounds require stringent management, including regular observation, assessment, documentation, and care of the wound by a medical professional. This management is tedious for patients as they need to visit the clinic frequently for specialist consultation, wound documentation, and adjunct therapies [1]. Furthermore, it strains the health care system, as it requires considerable clinical as well as financial resources. Repetitive wound documentation is tedious but essential to chronic wound care [2]. Treatment plans and scheduling of follow-up assessments are based on the information found in the wound documentation. Wound information such as wound location, depth, size, edges, and surrounding skin conditions is often documented, but can vary in detail among different clinical practices [2]. Therefore, the introduction of digitalization of chronic wound management to reduce the burden for patients, clinicians, and the health care system is desirable.

As part of the digitalization of chronic wound care, remote assessment of chronic wounds using mobile technology is raising great interest. Telemedicine approaches could reduce the constraints of time and geographical location and therefore reduce trips to clinics. It has been shown that connecting home-care nurses to hospital-based wound experts can significantly improve the likelihood of wound healing [3] and patient outcomes [4]. Patients sending wound images and symptom questionnaires enables remote follow-up monitoring for post-surgical wounds [5]. Smartphone apps with visual wound analytics and feedback to engage patients in self-care of diabetic foot ulcers resonated positively with the users in a usability study [6]. This study did not evaluate the accuracy of the automated wound image analysis. However, it highlighted the need for user-friendly image acquisition methods that assist the user in controlling the factors influencing the wound image quality.

Wound images constitute an essential part of chronic wound documentation during routine clinical assessment. Clinicians consult the images to assess wound changes [7]. Wound images are also used to determine wound dimensions and tissue conditions by measuring the wound area and color change over time [8]. However, current documentation approaches depend largely on routines established in the clinics, which are rarely standardized across institutions. Wound images are primarily taken by clinical staff with digital cameras and manually uploaded to the clinical information system to perform basic assessments, such as checking the wound history [7] and measuring the wound area [9].

The quality of wound images is essential for further analysis and processing. In clinical practice, high-quality images, characterized by attributes such as proper lighting condition and wound positioning, are prerequisites for wound experts to perform the wound assessment accurately and reliably, including visually inspecting the development of wound status and measuring the wound area from an image [10]. For automated image analysis, high-quality images, with characteristics such as proper lighting condition, corrected color, adequate sharpness, clear wound boundary, and often, the use of an associated color and size reference, are important for algorithms to perform specific tasks such as wound segmentation [11] or wound classification [12] with good performance. Specifications of cameras on modern smartphones are sufficient to be used in clinical practice [13]. However, image quality is largely based on environmental conditions and the person who captures the image. In mobile health (mHealth) applications, remote sensing and documentation by nonexperts are unsupervised and often prone to noise and artifacts [14]. For example, the reliability and accuracy of a teledermatoscopy-based diagnosis increased when the image quality improved [15]. Specifically, it is important to reduce blurriness and keep environmental conditions such as image angle and lighting consistent without over- or underexposure when capturing wound images, so that the wound size can be reliably calculated and the colors of wound areas can be correctly defined [16]. It is challenging to control the aforementioned conditions in a remote setting and with smartphones, especially for patients with little prior knowledge of technical and clinical requirements for wound images. Neither current clinical practices nor existing apps for remote wound monitoring have standardized procedures implemented for taking high-quality wound images. To our knowledge, no quality assurance measures for image quality have been proposed. In studies, low-quality images that are not useful are simply discarded and excluded from the analysis [16]. Therefore, it is crucial for mHealth systems that support remote documentation of chronic wounds to facilitate the high-quality acquisition of images that is sufficient for both visual clinical interpretation and automated processing.

Our goal was to develop an mHealth tool to facilitate the remote assessment of digital ulcers (DUs) and support patients with systemic sclerosis (SSc) in the self-management of their DUs. The tool should provide functions to self-document their wounds at home, including an image acquisition system that can ensure clinical-grade data quality. Such a system would enable telemedical functions for clinicians and reduce the burden of travel to the clinics for the patients.

### Systemic Sclerosis and Digital Ulcers

SSc is a systemic autoimmune disease characterized by fibrosis and microangiopathy. DUs are common in SSc, with an occurrence probability of up to 70% at a 10-year observation period [17]. Between one-third and two-thirds of patients with SSc develop recurrent DUs [18]. DUs are slow to heal and may complicate with bone infection and amputation, which significantly influence the patient’s quality of life and hand function [19]. The management of SSc-associated DUs often requires repeated presentation in a specialized clinic for assessment and adapted treatment, which may include wound debridation and specific topical measures. For this, patients face considerable burden from traveling to the clinics at a fixed time interval, and a timely assessment is not always achieved. There is a great unmet need for patients with SSc and DUs to be able to document their wounds remotely, in order to facilitate close follow-up by clinicians, enabling early detection of complications and reducing the burden induced by unnecessary travel to the clinic.

### Digital Health Tools for Wound Assessment

Many studies have investigated digital health tools for the assessment of wounds. Overall, these studies highlight the feasibility of remote wound care and self-documentation. However, only one study has evaluated the remote assessment of DUs so far [20]. Patients with SSc were using their own smartphone camera to take images of the DUs for a maximum of 35 days at home. While the study demonstrated the feasibility of home-based documentation of DUs, it was limited to 4 patients. No information on image quality was obtained, and images were manually collected and transferred to a database.

A large proportion of existing wound imaging and documentation apps focus on the assessment of diabetic foot ulcers. Yap et al [21] presented a mobile app aiming to standardize diabetic foot images by creating an outline of a precaptured foot image and aligning the foot when taking a new image. The images were taken in a clinical environment and it was up to the medical professional to decide whether to save the image, which might be challenging to translate to patient self-assessment. Patients are confronted with high uncertainty about the status of their own wound and might not be aware of the important parameters to control during wound image acquisition. Several apps integrate semi-automated wound measurement algorithms providing wound size and diameter [6,9]. However, the wound measurements require manual localization of the wound. Little evidence exists on the accuracy of such systems, due to the absence of validation studies on real wounds. To test interrater variability, plastic wound models have been used [9].

Besides an image-capturing app, supporting infrastructure is needed to enable ease of use and good-quality imaging results. The use of reference markers for color and size normalization is common [9]. Wang et al [22] developed a capturing box to capture wounds on feet that are otherwise not easily accessible. The box contains a mirror, and patients place their foot on a glass plate next to the phone that captures the mirror reflection inside the box. Alternatively, the front-facing camera can be used together with voice commands [6]. Neither of these studies investigated objective image quality of the obtained wound images, in particular when obtained by a nonmedically trained user.

We developed an mHealth tool to enable wound self-documentation by patients with SSc and DUs. Our specific aims of this study were to (1) define objective measures for assessing the quality of wound images taken by patients using this tool unsupervised at home, (2) compare whether an automated real-time feedback feature on wound image quality improves the quality of the transmitted wound images, and (3) evaluate the feasibility of implementing this mHealth tool for home-based chronic wound monitoring and self-management by patients with SSc.

## Methods

The newly developed mHealth tool was composed of a smartphone with a custom wound imaging and management app, a custom color reference sticker, and a smartphone holder. All components were designed to facilitate standardized wound image acquisition that is consistent over time and does not require supervision or advanced training of the user. With a randomized study design in a home-based setting, we tested and compared 2 versions of the smartphone app (basic and with feedback) that differed in the way the app interacts with the patient during image acquisition. The version with feedback relied on real-time algorithms to assess image quality.

### mHealth Tool Development

#### Wound Management App

**Figure 1:**
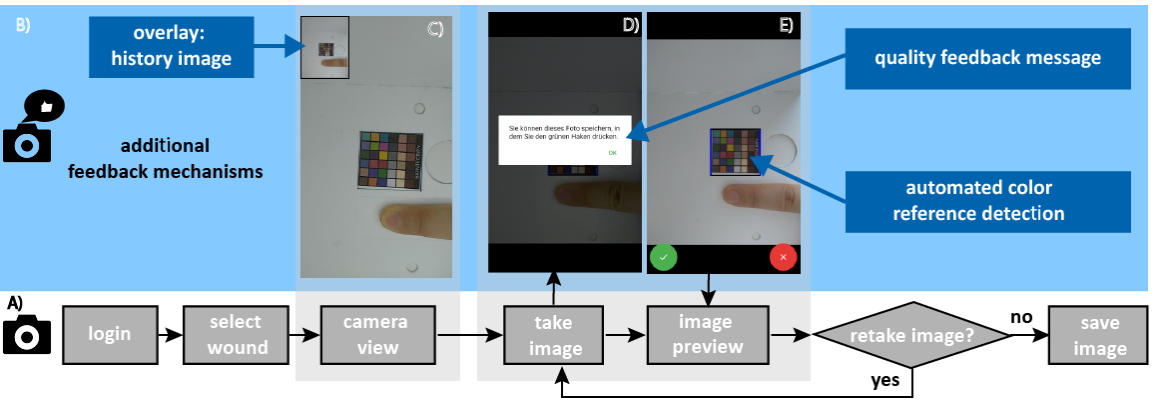
Flowchart of the interaction steps for a user for (A) the basic app and (B) additional feedback mechanisms for the version with feedback, which is illustrated with screenshots showing (C) a history image in the left corner of the screen to guide patients to create consistent fields of view across assessments, (D) feedback messages based on the automated detection of image quality, and (E) automated color reference detection to determine the image quality.

The smartphone app implemented a simple workflow to take wound pictures (Figure 1). It essentially consisted of a first-time patient login; first-time metadata entry (ie, wound locations), which was replaced with a selection in subsequent use; image acquisition; and background process that included secure upload to a REDCap study database [23] via an application programming interface (API) when an internet connection was available. The app was developed for the Android operating system. Two versions of the app were developed with the goal of improving the overall image quality. The basic app version did not provide any feedback on image quality other than showing the freshly acquired image for subjective review, which is native to the Android operating system. The version with feedback included specifically developed mechanisms to interact with the user and communicate quality improvement opportunities at the time of image acquisition (Figure 1).

#### Quality Feedback Mechanisms

For developing the feedback mechanisms, an objective measure for image quality was needed. We focused on the evaluation of image sharpness as it is a parameter that also measures how well the object of interest is in focus and has traditionally been an important parameter in image quality evaluation in other areas of application. Sharper images contain finer details and lead to more reliable object recognition and classification [24]. Blurry images negatively influence the performance of automated image processing algorithms [25]. In addition, it is recommended to discard blurry wound images and not use them for wound assessment, as they could mislead diagnosis or alter derived conclusions [16]. Therefore, it was desirable to identify unsuitable images early in the process and encourage repetition of the image acquisition at the instance where the user is already engaged with the process. For this purpose, we defined the sharpness as the variance of the Laplacian [26]. The Laplacian is a second derivative operator to high-pass spatial frequencies, which are associated with sharp edges, and it is sensitive to the rapid intensity changes in an image [26]. The variance reflects the spread of intensity changes. Thus, sharper images have higher Laplacian variance.

An additional objective measure for quality available in our system was the desired presence of the color reference sticker in the wound image. Therefore, we implemented an automatic color reference sticker detection routine into the imaging process. First, the color checker position was detected in the field of view by analyzing the rectangular morphology and exceeding a predefined threshold of its size, and then the sharpness of the sticker was analyzed. When the color reference sticker was detected and the corresponding region of interest was deemed sharp enough by exceeding a predefined threshold, the app provided the patient with a feedback message indicating that the image is deemed to be of good quality and can be saved. Otherwise, the user was prompted to retake the image.

As a third feedback mechanism, we provided a subjective comparison between the current image preview and a previously taken image of the same wound. The historical image was displayed in the left corner of the image preview screen. The intention of this was to encourage the patient to maintain a consistent field of view across multiple capturing sessions.

#### Color Reference Sticker

We developed a customized color reference sticker as part of a larger project named SwissWOU, which is a subproject of the SKINTEGRITY Flagship of the Universities and Hospitals of Zurich, with the aim of developing a large wound image database for wound research. The color reference sticker contained 36 color patches that included all the colors for regular photography color calibration [27] and an additional set of skin and wound-specific color shades (total size 30 x 30 mm).

#### Smartphone Holder

**Figure 2:**
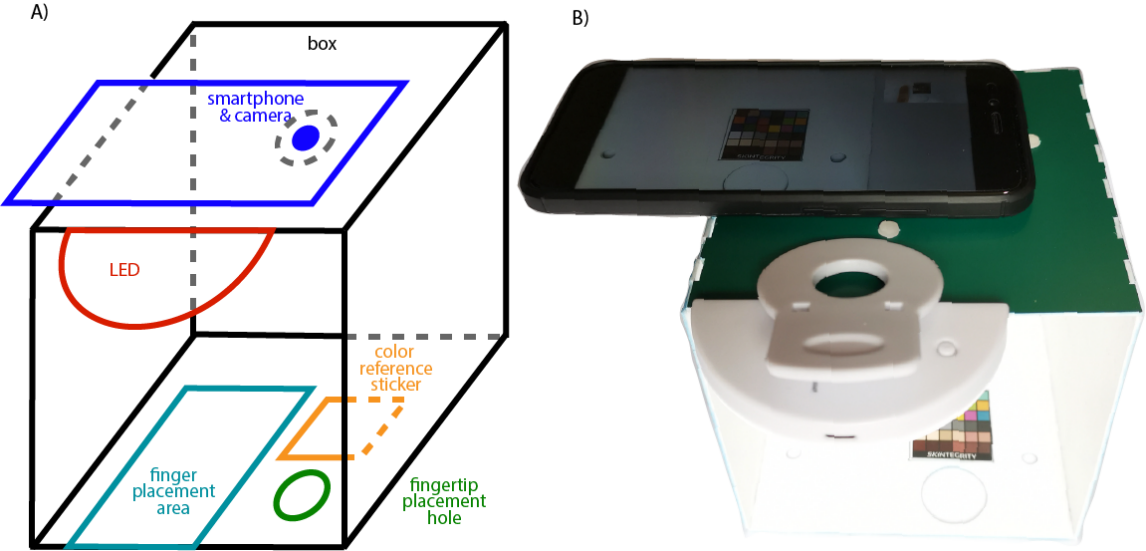
(A) 3D schematic of the mobile health tool and (B) experimental setup.

Patients with SSc often have severely reduced hand and finger function [19], limiting the range of motion and possibilities to interact with a smartphone. To ease the image-taking procedure and address the patient’s needs, we designed a smartphone holder (Figure 2). Patients placed the smartphone on top of the smartphone holder. Their finger was placed on the bottom plate or, in the case of fingertip wounds, through a hole in the bottom plate. The color reference sticker was also placed on the bottom plate adjacent to the finger placement area. An additional LED light ring assured a homogeneous lighting condition and prevented strong shadows. The smartphone holder provided a constant distance between the camera and the wound and consistent illumination, thereby also contributing to the consistency of image quality across time series.

### Experimental Protocol

The study protocol followed the ethical principles for research involving humans according to the Declaration of Helsinki and was approved by the institutional ethics committee (ETH EK 2019-N-22). We recruited consecutive patients with SSc who attended the Department of Rheumatology at the University Hospital Zurich, Switzerland, for routine or emergency consultations from May 2019 to May 2020. Adult patients fulfilling classification criteria for SSc [28] and having at least one DU on the fingers were included after written informed consent. First, patients performed a practical baseline test and filled in a questionnaire on mobile literacy and familiarity with smartphone camera usage. We identified patients who were familiar with using basic smartphone functions and also patients who were accustomed to photography. Based on this test, we stratified patients into experienced users and nonexperienced users, based on the combined criteria of whether they used the smartphone multiple times a day or used it at least once a day and were familiar with taking photos or videos. We randomly assigned the patients to 2 device groups (feedback and basic groups), while balancing the user experience between the groups. After this randomization, all patients were given an Android smartphone with all required apps pre-installed and the smartphone holder to keep for the duration of the study. We then instructed them on how to use the mHealth tool, explained the study protocol, and walked them step by step through the image-taking process of the corresponding app version (feedback or basic) once. Questions from patients were then answered. Under supervision, the patients then used the tool by themselves to take the first set of images of their wounds.

**Figure 3:**
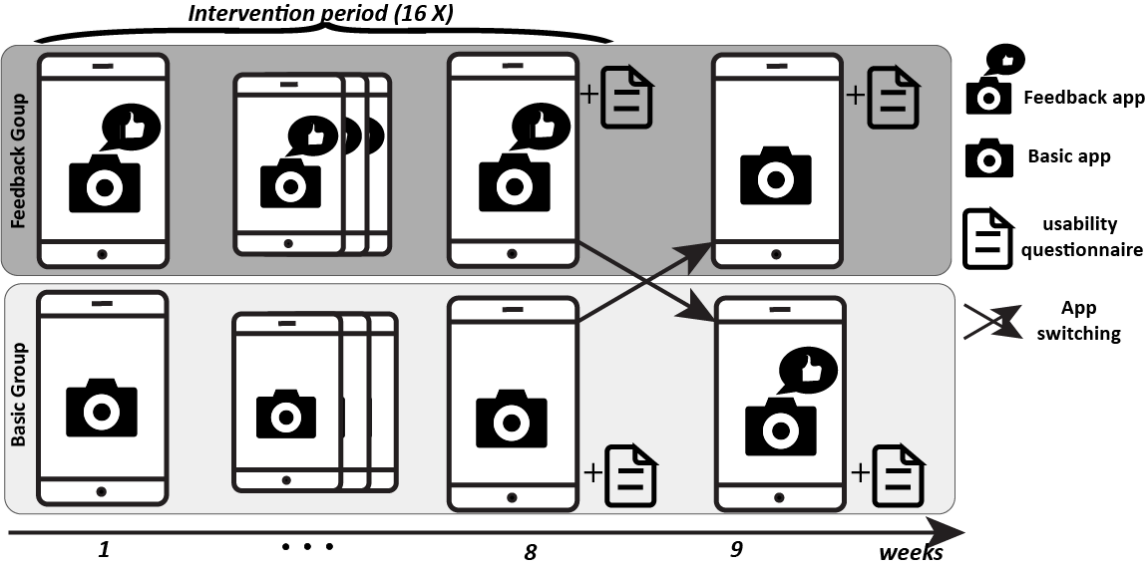
Overview of the wound assessment protocol performed at the patient’s home for both the feedback and basic groups, which involved wound assessments every third day (16 times in total) for the first 8 weeks, after which, patients completed the usability questionnaires. In week 9, patients switched to the alternative app version and performed image assessments every third day (2-3 times), after which they evaluated the usability.

The data acquisition at home followed a regular protocol (Figure 3). Both groups were asked to perform the wound assessments with the app every third day for the duration of 8 weeks (16 assessments). The assessment consisted of taking wound images and completing pain level questionnaires. The patient had to first login to unlock the app, place the smartphone on the smartphone holder, turn on the LED light ring, select the wound location, take an image, and if it was deemed of good quality, save the image or otherwise retake an image. After completing the wound image capture, patients evaluated their pain levels. At the end of the 8th week, data collection for the randomized app and image quality evaluation was completed, and a usability questionnaire was delivered. For an additional week on every third day (2-3 assessments), patients got to explore and test the alternate app version from the other group and evaluate the usability of this version as well. At the end of the study, patients returned the mHealth tool by mail.

### Analysis

#### Image Quality Comparison

To evaluate and compare the image quality between the randomized groups, we calculated the following image quality parameters.

We defined the color checker detection ratio (CCDR) as the number of images with clearly visible color reference stickers detected over the total number of images collected (Equation 1). We calculated the CCDR across each group and patient



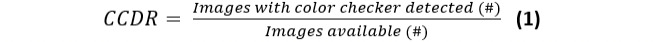



We compared the sharpness of images between groups, where the variance of the Laplacian was selected as a measure for sharpness [26]. Since the variance of the Laplacian is dependent on the image content and the variability of wounds across subjects was not negligible, we restricted the region of interest to the color reference sticker, which was expected to be available and constant across all wound images. We evaluated the color checker sharpness (CCS) of each image where the color reference sticker area was manually labeled by a researcher after the data acquisition. The median CCS for each patient was evaluated to compare between groups.

In addition to the above-calculated quality measurements, subjective image quality was obtained. A research assistant was trained to evaluate the quality of wound images. She labeled all the images blinded to the randomized groups and applied a binary label. The binary label indicated whether the image was usable for the unambiguous identification of the finger and wound area. The ratios of usable images across each group and patient were calculated.

#### Statistical Analysis of Image Quality Comparison

We performed a Wilcoxon rank-sum test to compare whether there were statistically significant differences in the image quality between the 2 groups for the images that were collected from the first 8-week documentation period. The analysis was carried out with the Python SciPy library (version 1.4.1) [29]. A *P* value <.01 was considered statistically significant.

#### Feasibility Evaluation

The feasibility of the mHealth intervention was determined by analyzing the usability feedback from the questionnaires, the user behavior throughout the study, and the overall quality of wound images as described in the previous section.

The patients rated the usability of the mHealth tool with a questionnaire after each type of use (basic or feedback app) had ended. The questionnaires consisted of questions on the overall mHealth tool experience and the subcomponents like the smartphone holder and the versions of the app. The usability questions originated from the Post-Study System Usability Questionnaire [30], which is widely used to measure users’ perceived satisfaction, such as software, system, or product at the end of a study, and were adapted to the specific functions of our application. Additionally, we asked questions about whether the patients were willing to continue using the tool and which app version they preferred. Each question was answered on a 7-point Likert scale, where 1 indicated “strongly agree” and 7 indicated “strongly disagree.” For analysis, we aggregated the answers in bar plots.

The monitoring of user behavior was focused on whether the patients (1) were able to self-document their wound images and follow the study protocol with good adherence, (2) were efficient in using the app to capture images, and (3) dropped out of the study. The self-documentation was evaluated based on the patients’ compliance with the image documentation routine given by the protocol. We calculated the number of image capturing assessment days actually performed divided by the assessment days that were assigned to be performed during the 8 weeks (16 assessments). When a patient took more days of assessments than expected (eg, ratio higher than 1), we then assigned the ratio as 1. We evaluated the efficiency by calculating the duration of each assessment by measuring the time between starting the app and the end of the assessment. For patients who documented multiple wounds, we only measured the time until saving the first wound image. We eliminated duration outliers from the analysis as they may have resulted from other distractions unrelated to the imaging task. Outliers were defined as a measurement duration of more than 3 scaled median absolute deviations away from the median duration over all assessments from each patient. For each group, we then calculated median durations and fitted a linear trend over time to evaluate the assessment efficiency.

## Results

A total of 21 patients were recruited and randomized into feedback (10 patients) and basic (11 patients) groups. During the course of the study, 6 patients returned the mHealth tool before completing the protocol and were subsequently excluded from the image quality analysis (Table 1). All dropouts happened in the first 2 weeks (Table 2) and were by female participants (Table 1). They had balanced strata (Table 1), and 2 belonged to the feedback group and 4 to the basic group (Table 2). The reasons for not continuing with the study were severe illness (3 patients), overwhelmed after instructions (2 patients), and overwhelmed after subsequent usage (1 patient; Table 2). Of the 15 patients who completed the study, 8 were in the feedback group, and 7 were in the basic group. All of the included patients were in the experienced smartphone user group strata (Table 1). A total of 382 wound images were collected during the intervention period, and 24 images were collected during week 9 after switching apps for the second usability evaluation (Table 1).

**Table 1:** Metadata for the patient randomization, dropouts, and number of images from each group.

Patient characteristics			Intervention period (Weeks 1-8)				Week 9					Dropouts (n=6)
			Feedback (n=8)		Basic (n=7)		Feedback (n=4)		Basic (n=4)			
Age (years), mean (SD)			47 (11)		52 (9)		N/A^a^		N/A		48 (13)	
**Sex, n**												
	Male	4		1		N/A		N/A		0		
	Female	4		6		N/A		N/A		6		
**Strata ratio^b^, n**												
	Not familiar	0		0		N/A		N/A		3		
	Familiar	8		7		N/A		N/A		3		
Total number of images			199		183		15		9		N/A	
Distribution of images per patient, mean (SD)			24.88 (11.21)		26.14 (10.59)		1.88 (1.76)		1.29 (1.39)		N/A	
Number of images with color checker detected			191		158		N/A		N/A		N/A	
Subjective quality^c^, n			180		155		15		7		N/A	

^a^N/A: not applicable.

^b^Ratio of the number of patients that were not familiar to those who were familiar with smartphone camera usage.

^c^Number of images manually labeled as good quality by the research assistant.

**Table 2:** Study dropouts and time since recruitment, by dropout reason.

Patient characteristics		Overwhelmed at instruction (n=2)	Severe illness (n=3)	Overwhelmed during usage (n=1)
Time since recruitment for each patient (days)		0, 0	3, 6, 9	13
**Strata ratio^a^, n**				
	Not familiar	1	2	0
	Familiar	1	1	1
**Group, n**				
	Feedback	1	0	1
	Basic	1	3	0

^a^Ratio of the number of patients that were not familiar to those who were familiar with smartphone camera usage.

### Image Quality Comparison

#### Color Checker Detection Ratio

**Figure 4:**
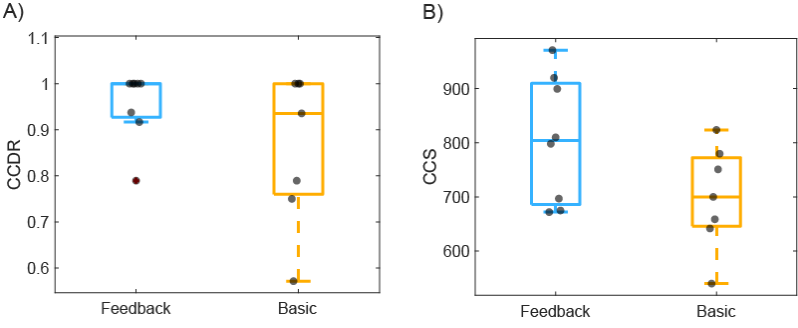
(A) Color checker detection ratio (CCDR) and (B) color checker sharpness (CCS) per patient for the feedback and basic groups. The grey dots indicate each individual patient. The central, bottom, and top edges of the boxes indicate the median, 25th percentile, and 75th percentile, respectively; the whiskers indicate the outliers.

The CCDR was 0.96 (191/199) in the feedback group compared to 0.86 (158/183) in the basic group. As depicted in the boxplot in Figure 4, the feedback group faced less variance (median 1; 25th percentile=0.93; 75th percentile=1) compared to the basic group (median 0.94; 25th percentile=0.76; 75th percentile=1).

#### Color Checker Sharpness

**Figure 5:**
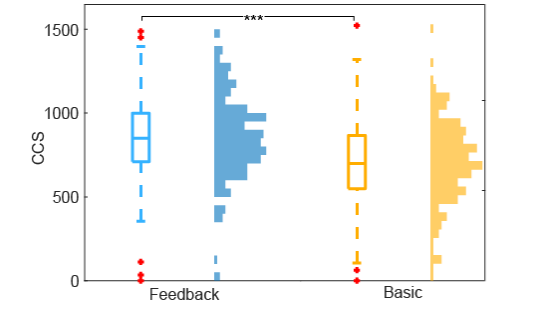
Comparison of the color checker sharpness (CCS) distribution for all images between the feedback and basic groups. The central, bottom, and top edges of the boxes indicate the median, 25th percentile, and 75th percentile. ***P<.001.

The feedback group achieved higher median CCS across patients (median 804) compared to the basic group (median 700; Figure 4). The feedback group also showed overall higher CCS distribution across all images (median 894; 25th percentile=710; 75th percentile=999 ) compared to the basic group (median 700; 25th percentile=549; 75th percentile=867) as depicted in Figure 5. The Wilcoxon rank-sum test showed a significant difference (*P*<.001) between the 2 groups.

#### Manually Labeled Image Quality

**Figure 6:**
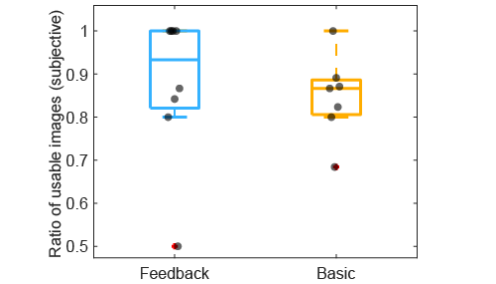
Distribution of subjective image quality, which was measured as the ratio of images that were labeled as usable, for the manually labeled images for the feedback and basic groups. The grey dots indicate each individual patient. The central, bottom, and top edges of the boxes indicate the median, 25th percentile, and 75th percentile, respectively.

In the feedback group, 90% (180/199) of the images were subjectively labeled as usable compared to 85% (155/183) in the basic group (Table 1). The feedback group had a higher ratio of images per patient that were manually labeled as usable (median 0.93; 25th percentile=0.82; 75th percentile=1) than the basic group (median 0.87; 25th percentile=0.81; 75th percentile=0.88) as depicted in Figure 6.

### Feasibility Evaluation

**Figure 7:**
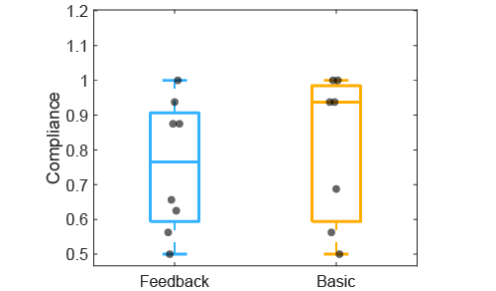
Ratio of patients’ compliance to the documentation routine for the feedback and basic groups. The grey dots indicate each individual patient.

The median ratios of the patients’ compliance to the image documentation routine were 0.77 (25th percentile=0.59; 75th percentile=0.91) for the feedback group and 0.94 (25th percentile=0.59, 75th percentile=0.98) for the basic group (Figure 7). The overall median compliance ratio for both groups was 0.88 (25th percentile=0.58; 75th percentile=0.94)

**Figure 8:**
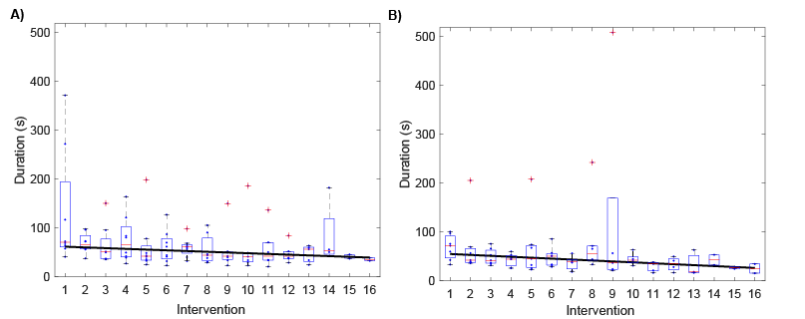
Durations of image assessments over the 8-week study period, consisting of 16 interventions for the (A) feedback and (B) basic groups. The solid lines are the linearly fitted trends per group.

The median durations for one image assessment were 42 seconds (overall), 47 seconds (feedback group), and 42 seconds (basic group). The assessment durations showed a decreasing trend for both the feedback and basic groups (Figure 8).

**Figure 9:**
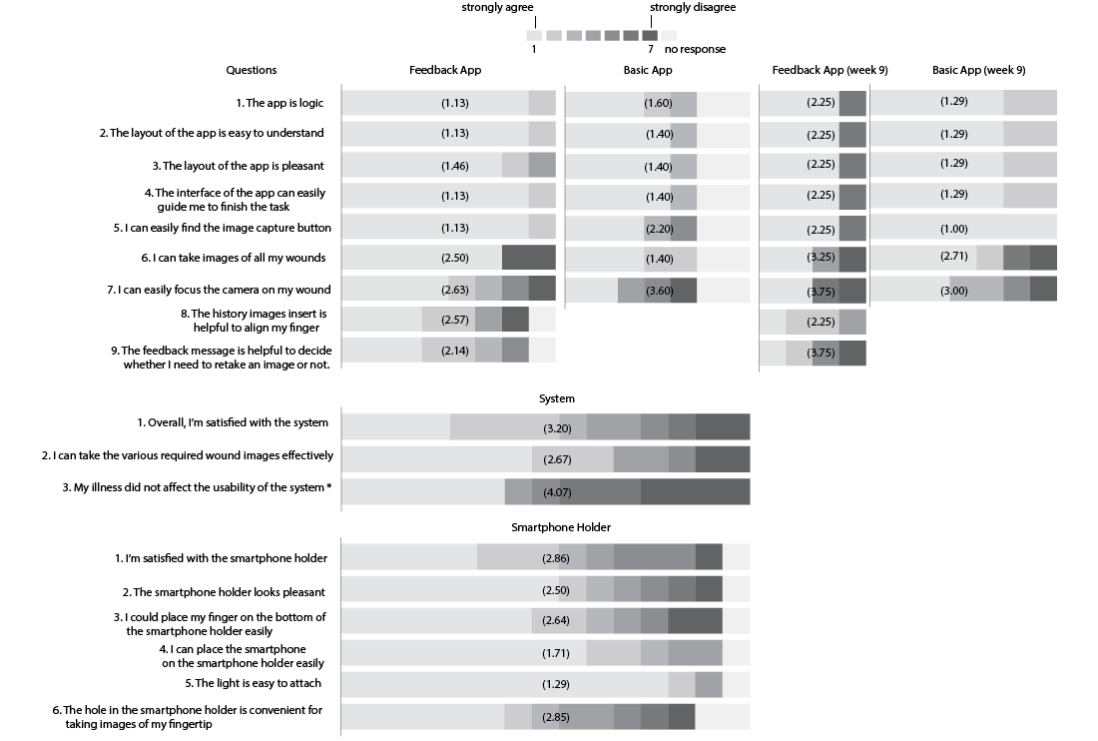
Results of the usability evaluation, in which each question was answered on a 7-point Likert scale (1=strongly agree, 7=strongly disagree). The average rating is shown in brackets on the barplot. *The 3rd question from “System” and its rating were reversed from its original form (Does my illness affect the usability of the system?).

The usability rating showed an overall higher user agreement for the feedback app (range 1.13-2.63) compared to the basic app (range 1.40-3.60) after the 8th week (Figure 9). The smartphone holder obtained an agreement between 1.29 and 2.86 for both groups, and the mHealth tool as a whole was rated between 2.67 and 4.07. At week 9, after switching the app version, the feedback group rated the basic version app (range 1.00-3.00) similarly to the previously used feedback app (Figure 9). The basic group rated the feedback version app lower (range 2.25-3.75) than their scores for the basic version app (Figure 9).

**Figure 10:**
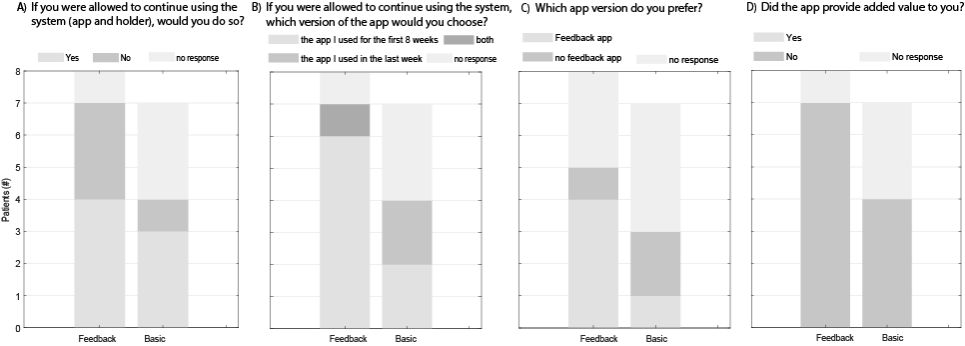
Additional questionnaire with 4 questions about (A) the patients’ willingness to continue using the system, (B,C) their preference on the app version, and (D) whether the app added value for them.

A total of 7 patients indicated an interest in using the system in the future (Figure 10). They provided several reasons:

…be able to communicate changes without going to the hospital to check.

…provide information to support wound healing.

…monitor how the wound is changing and to be able to intervene more quickly…

The responses from the 4 patients who answered “no” were:

…only when I feel that my condition is getting worse.

I don’t see what to use it for.

I would find a solution that is easier for me.

Because not all wounds can be photographed.

Of the 8 patients in the feedback group, 6 preferred the feedback app over the basic app (Figure 10), while 2 of 4 patients from the basic group preferred the basic app.

## Discussion

### Principal Findings

We developed an mHealth tool for SSc patients with DUs to remotely self-document wound images in a standardized way and introduced an image quality–based feedback mechanism to encourage higher data integrity through targeted human-machine interaction. To our knowledge, this was the first time that a research study systematically evaluated the quality of patient self-documented wound images with a randomized protocol. We introduced objective image quality parameters such as CCDR and CCS that use the standardized region of interest of a color reference sticker to enable a reproducible quality estimation. These objective image quality metrics, along with subjective expert image assessments, showed that using the feedback mechanism yielded a higher image quality compared to using the basic app without real-time feedback. More specifically, the CCDR, CCS per patient, and manually labeled image quality were higher for the feedback group compared to the basic group. The feedback group achieved significantly higher CCS over all image distributions compared to the basic group. Overall, the high number of good-quality images obtained and the usability evaluation indicated that home-based DU assessment by SSc patients who are familiar with digital tools is feasible.

The importance of taking high-quality wound images for diagnostic and treatment monitoring purposes cannot be emphasized enough. From a technical point of view, modern smartphones are designed to capture high-resolution images with color representations comparable to professional digital cameras [13]. However, capturing images of wounds for clinical applications is more critical and has more stringent requirements, because bad image quality could negatively influence the diagnosis and management plan [15]. With our automatic feedback mechanism that processed images in real-time at the smartphone frontend, we improved the image quality during the process of image collection. This approach increased the reliability and consistency of images and avoided postprocessing or discarding of poor images, therefore also increasing the integrity of the data. Such an increase could positively impact further processing of the images, such as automated classification and segmentation algorithms or assessments of abnormalities during telemedical consultations. Whether the increase in quality has an impact on such applications and can influence patient health outcomes need to be investigated in a separate study.

We expected to observe a benefit from the real-time feedback mechanism. Other digital health applications previously reported such benefits. For example, respiratory rate counters that provide audio and visual feedback enable direct comparison of measurements with the breathing subject. Such comparison leads to improved respiratory rate estimations and repeated measurements where no initial agreement between feedback and subjects can be observed [31]. Furthermore, computing a signal quality index that is displayed in real time with a color coding in the background of a vital sign trace assists the user in recording good-quality pulse oximetry recordings [32]. In fact, specifications in standards for medical pulse oximeters (ISO 80601-2-61) enforce a real-time indication for signal inadequacy [33]. However, standards leave it up to the manufacturer to define the quality indicator metric and its display method. Therefore, it is important to test its performance, usability, and feasibility in a user study as part of the medical device validation and certification process. The proposed randomization of users in 2 groups that use 2 different mHealth tools is a direct way to evaluate which tool can provide better quality data.

The usability questionnaires were another way to evaluate the feasibility and showed that the majority of the patients were satisfied with the system. All questions except one received a rating ≤3.75 on a 7-point Likert scale. The question of whether the usability of the system was influenced by the patient’s illness received a rating of 4.07. A closer investigation of this result revealed 2 reasons that should be considered in further designs. First, hand function of some patients was severely impacted by the disease. Two patients brought forward that it was not easy to place their fingers correctly in the smartphone holder box to capture all angles of the wounds. Second, one patient stated that it was more difficult to get a sharp image from specific wound locations. This indicates that there is still room for improvement of the mHealth tool design. For patients whose hand function is severely impaired by the disease or whose fingers are affected on both hands, an obvious solution to avoid additional painful interaction with the system could be assistance from a second person (eg, family member or home care staff) or the integration of voice commands, which has already been proposed for diabetic foot ulcer management [6]. Additional technical developments might be required for this (ie, an algorithm for the automated election of the focus point).

We re-evaluated the usability after switching the app version and using the alternative for another week. Interestingly, the feedback group did provide a similar usability rating for the basic app version, while the basic group had lower usability ratings for the feedback app version. An explanation could be that the basic group did not receive training on the feedback mechanisms, which might have led to confusion and lower usability. This would suggest that adequate training is needed before using the mHealth tools and could lead to better acceptance of the intervention.

We reported 6 patients who dropped out during the course of the study. The dropouts occurred immediately after recruiting or at an early stage of the study. The main reason for dropout was due to severe illness and the need for hospitalization, so that the assessment had to be stopped. It is interesting to note that all 3 subjects that were placed in the “not familiar with smartphone use” strata left the study prematurely. While this is too small a number to draw conclusions, it may indicate that familiarity with smartphone use could be a prerequisite for patients to engage with an mHealth approach to wound documentation. This important parameter, together with low recruiting rates, should be closely monitored in subsequent studies and analyzed to determine whether they indicate that the use of technology might not be suitable for all patient groups or demographics.

We demonstrated that remote self-management of wounds by patients with SSc is feasible. An overall rate of routine documentation of 88% was achieved by patients, indicating good adherence to the suggested protocol. Independent of the app version, the obtained image quality was sufficient for further use (>84% of images got a usable label from the subjective expert assessment). The median image assessment duration was within the range of 50 seconds, indicating that the mHealth tool is efficient to use and can be integrated into the daily routine of a patient. While our analysis was limited to 15 patients, this number was sufficient to highlight the preferences of participants and demonstrate the benefits of real-time feedback on improving wound image quality. The usability results indicated that, with small adaptations and optimizations of the design, satisfaction of the patients could be further improved. With 64% of the patients willing to continue using the current version of the mHealth tool, these adaptations will be well justified.

The technical features of the mHealth tool could also be improved in further design iterations. Currently, the image-taking process was based on the standard camera sensor of the smartphone, which produced 2D RGB images that can be used for documenting the wound and assessing basic wound characteristics such as area and color. More advanced measuring approaches could involve the recording of 3D wound images for wound depth assessment [34] or using additional sensor modalities, such as a thermal or infrared sensor to visualize the perfusion changes in and around a wound [35]. Such modalities would be even more difficult to subjectively assess for quality by a lay user because they are less common and require more advanced user interaction. Therefore, integrating automated quality mechanisms would be of utmost importance also in these approaches. However, different objective metrics will need to be developed.

The impact of a home-based mHealth tool for self-documentation of wounds and telemedical application for patients with SSc was evident. First of all, patients identified that such tools could reduce unnecessary travel to the clinics. In addition, during the COVID-19 pandemic, patients with SSc were considered more vulnerable to infection due to immunosuppressive treatment that dampens the immune system [36]. Therefore, patients with autoimmune diseases were recommended to avoid crowds and unnecessary travel or hospital visits [36]. In such a context, telemedicine consultations could serve as a valid tool for enhancing disease management without additionally endangering the patients. No less important, the transmission of wound images with the mHealth tool could enable clinicians or decision support algorithms to detect abnormal wound conditions, such as new or worsening ulcers, earlier [37], leading to more timely referral and earlier treatment.

### Conclusions

There is a great need for mobile solutions to support self-documentation of chronic wounds, as well as to enable clinical-grade wound image acquisition at the patient’s home. We developed an mHealth tool that provides such health services and enables telemedical support for patients with SSc and DUs. We demonstrated that it is feasible to deploy such an app in this patient group and high-quality wound images can be consistently acquired. System usability had positive ratings, with room for improvement to address the disease-specific needs of patients. Introducing a feedback mechanism identifying image quality deficiencies and encouraging repetition of the imaging process improved overall image quality when compared to a solution without such a feedback mechanism. The mHealth tool can be further investigated to evaluate the clinical efficacy and effectiveness and establish whether patient outcomes could be improved with this sensor-based telemedicine intervention.

## Abbreviations

API: application prorgramming interface
CCDR: color checker detection ratio
CCS: color checker sharpness
DU: digital ulcer
mHealth: mobile health
SSc: systemic sclerosis

## References

[1] Othman D (2012). Negative pressure wound therapy literature review of efficacy, cost effectiveness, and impact on patients' quality of life in chronic wound management and its implementation in the United kingdom. Plast Surg Int.

[2] Khalil H, Cullen M, Chambers H, Steers N, Walker J (2014). Implementation of a successful electronic wound documentation system in rural Victoria, Australia: a subject of collaboration and community engagement. Int Wound J.

[3] Zarchi K, Haugaard VB, Dufour DN, Jemec GBE (2015). Expert advice provided through telemedicine improves healing of chronic wounds: prospective cluster controlled study. J Invest Dermatol.

[4] Vowden K, Vowden P (2013). A pilot study on the potential of remote support to enhance wound care for nursing-home patients. J Wound Care.

[5] Segura-Sampedro JJ, Rivero-Belenchón I, Pino-Díaz V, Rodríguez Sánchez MC, Pareja-Ciuró F, Padillo-Ruiz J, Jimenez-Rodriguez RM (2017). Feasibility and safety of surgical wound remote follow-up by smart phone in appendectomy: A pilot study. Ann Med Surg (Lond).

[6] Ploderer B, Brown R, Seng LSD, Lazzarini PA, van Netten JJ (2018). Promoting Self-Care of Diabetic Foot Ulcers Through a Mobile Phone App: User-Centered Design and Evaluation. JMIR Diabetes.

[7] Hess CT (2005). The art of skin and wound care documentation. Home Healthc Nurse.

[8] Queen D, Harding K (2020). Is wound photography becoming sloppy?. Int Wound J.

[9] Wang SC, Anderson JAE, Evans R, Woo K, Beland B, Sasseville D, Moreau L (2017). Point-of-care wound visioning technology: Reproducibility and accuracy of a wound measurement app. PLoS One.

[10] Rennert R, Golinko M, Kaplan D, Flattau A, Brem H (2009). Standardization of wound photography using the Wound Electronic Medical Record. Adv Skin Wound Care.

[11] Goyal M, Reeves ND, Rajbhandari S, Yap MH (2019). Robust Methods for Real-Time Diabetic Foot Ulcer Detection and Localization on Mobile Devices. IEEE J. Biomed. Health Inform.

[12] Shenoy V, Foster E, Aalami L, Majeed B, Aalami O (2018). Deepwound: Automated Postoperative Wound Assessment and Surgical Site Surveillance through Convolutional Neural Networks. Cornell University.

[13] Boissin C, Fleming J, Wallis L, Hasselberg M, Laflamme L (2015). Can We Trust the Use of Smartphone Cameras in Clinical Practice? Laypeople Assessment of Their Image Quality. Telemed J E Health.

[14] Zhang J, Tüshaus L, Nuño Martínez N, Moreo M, Verastegui H, Hartinger SM, Mäusezahl D, Karlen W (2018). Data Integrity-Based Methodology and Checklist for Identifying Implementation Risks of Physiological Sensing in Mobile Health Projects: Quantitative and Qualitative Analysis. JMIR Mhealth Uhealth.

[15] van der Heijden JP, Thijssing L, Witkamp L, Spuls PI, de Keizer NF (2013). Accuracy and reliability of teledermatoscopy with images taken by general practitioners during everyday practice. J Telemed Telecare.

[16] Sperring B, Baker R (2014). Ten top tips for taking high-quality digital images of wounds. Wound Essentials.

[17] Wirz EG, Jaeger VK, Allanore Y, Riemekasten G, Hachulla E, Distler O, Airò P, Carreira PE, Tikly M, Vettori S, Balbir Gurman A, Damjanov N, Müller-Ladner U, Distler J, Li M, Häusermann P, Walker UA, EUSTAR coauthors (2016). Incidence and predictors of cutaneous manifestations during the early course of systemic sclerosis: a 10-year longitudinal study from the EUSTAR database. Ann Rheum Dis.

[18] Mihai C, Landewé R, van der Heijde D, Walker UA, Constantin PI, Gherghe AM, Ionescu R, Rednic S, Allanore Y, Avouac J, Czirják L, Hachulla E, Riemekasten G, Cozzi F, Airò P, Cutolo M, Mueller-Ladner U, Matucci-Cerinic M, EUSTAR co-authors (2016). Digital ulcers predict a worse disease course in patients with systemic sclerosis. Ann Rheum Dis.

[19] Hughes M, Herrick AL (2017). Digital ulcers in systemic sclerosis. Rheumatology (Oxford).

[20] Dinsdale G, Moore TL, Manning JB, Murray AK, Atkinson R, Ousey K, Dickinson MR, Taylor C, Herrick AL (2018). Tracking digital ulcers in systemic sclerosis: a feasibility study assessing lesion area in patient-recorded smartphone photographs. Ann Rheum Dis.

[21] Yap MH, Chatwin KE, Ng C, Abbott CA, Bowling FL, Rajbhandari S, Boulton AJM, Reeves ND (2018). A New Mobile Application for Standardizing Diabetic Foot Images. J Diabetes Sci Technol.

[22] Wang L, Pedersen PC, Strong DM, Tulu B, Agu E, Ignotz R (2015). Smartphone-based wound assessment system for patients with diabetes. IEEE Trans Biomed Eng.

[23] Harris PA, Taylor R, Thielke R, Payne J, Gonzalez N, Conde JG (2009). Research electronic data capture (REDCap)--a metadata-driven methodology and workflow process for providing translational research informatics support. J Biomed Inform.

[24] de Villiers JP (2010). A comparison of image sharpness metrics and real-time sharpening methods with GPU implementations.

[25] Dodge S, Karam L (2016). Understanding how image quality affects deep neural networks.

[26] Pech-Pacheco JL, Cristöbal G, Chamorro-Martínez J, Fernândez-Valdivia J (2000). Diatom autofocusing in brightfield microscopy: a comparative study.

[27] McCamy CS, Marcus H, Davidson JG (1976). Color-Rendition Chart. Journal of Applied Photographic Engineering.

[28] van den Hoogen F, Khanna D, Fransen J, Johnson SR, Baron M, Tyndall A, Matucci-Cerinic M, Naden RP, Medsger TA, Carreira PE, Riemekasten G, Clements PJ, Denton CP, Distler O, Allanore Y, Furst DE, Gabrielli A, Mayes MD, van Laar JM, Seibold JR, Czirjak L, Steen VD, Inanc M, Kowal-Bielecka O, Müller-Ladner U, Valentini G, Veale DJ, Vonk MC, Walker UA, Chung L, Collier DH, Csuka ME, Fessler BJ, Guiducci S, Herrick A, Hsu VM, Jimenez S, Kahaleh B, Merkel PA, Sierakowski S, Silver RM, Simms RW, Varga J, Pope JE (2013). 2013 classification criteria for systemic sclerosis: an American College of Rheumatology/European League against Rheumatism collaborative initiative. Arthritis Rheum.

[29] Virtanen P, Gommers R, Oliphant TE, Haberland M, Reddy T, Cournapeau D, Burovski E, Peterson P, Weckesser W, Bright J, van der Walt SJ, Brett M, Wilson J, Millman KJ, Mayorov N, Nelson ARJ, Jones E, Kern R, Larson E, Carey CJ, Feng Y, Moore EW, VanderPlas J, Laxalde D, Perktold J, Cimrman R, Henriksen I, Quintero EA, Harris CR, Archibald AM, Ribeiro AH, Pedregosa F, van Mulbregt P, Polat, SciPy 1.0 Contributors (2020). SciPy 1.0: fundamental algorithms for scientific computing in Python. Nat Methods.

[30] Lewis JR (2002). Psychometric Evaluation of the PSSUQ Using Data from Five Years of Usability Studies. International Journal of Human-Computer Interaction.

[31] Karlen W, Gan H, Chiu M, Dunsmuir D, Zhou G, Dumont GA, Ansermino JM (2014). Improving the accuracy and efficiency of respiratory rate measurements in children using mobile devices. PLoS One.

[32] Garde A, Zhou G, Raihana S, Dunsmuir D, Karlen W, Dekhordi P, Huda T, Arifeen SE, Larson C, Kissoon N, Dumont GA, Ansermino JM (2016). Respiratory rate and pulse oximetry derived information as predictors of hospital admission in young children in Bangladesh: a prospective observational study. BMJ Open.

[33] (2017). ISO 80601-2-61:2017 Medical electrical equipment — Part 2-61: Particular requirements for basic safety and essential performance of pulse oximeter equipment. International Standards Organization.

[34] Kuang B, Pena G, Szpak Z, Edwards S, Battersby R, Cowled P, Dawson J, Fitridge R (2021). Assessment of a smartphone-based application for diabetic foot ulcer measurement. Wound Repair Regen.

[35] Maddah E, Beigzadeh B (2020). Use of a smartphone thermometer to monitor thermal conductivity changes in diabetic foot ulcers: a pilot study. J Wound Care.

[36] Orlandi M, Lepri G, Bruni C, Wang Y, Bartoloni A, Zammarchi L, Cometi L, Guiducci S, Matucci-Cerinic M, Bellando-Randone S (2020). The systemic sclerosis patient in the COVID-19 era: the challenging crossroad between immunosuppression, differential diagnosis and long-term psychological distress. Clin Rheumatol.

[37] Hughes M, Allanore Y, Chung L, Pauling JD, Denton CP, Matucci-Cerinic M (2020). Raynaud phenomenon and digital ulcers in systemic sclerosis. Nat Rev Rheumatol.

